# α1-Syntrophin Variant Identified in Drug-Induced Long QT Syndrome Increases Late Sodium Current

**DOI:** 10.1371/journal.pone.0152355

**Published:** 2016-03-30

**Authors:** Jong-Il Choi, Chaojian Wang, Matthew J. Thomas, Geoffrey S. Pitt

**Affiliations:** 1 Division of Cardiology, Department of Medicine, Duke University School of Medicine; and Ion Channel Research Unit, Duke University Medical Center, Durham, NC, United States of America; 2 Division of Cardiology, Department of Internal Medicine, Korea University College of Medicine and Korea University Medical Center, Seoul, Republic of Korea; 3 Division of Genetics, Department of Pediatrics, University of Virginia Health System, Charlottesville, VA, United States of America; Xuzhou Medical College, CHINA

## Abstract

Drug-induced long-QT syndrome (diLQTS) is often due to drug block of *I*_Kr_, especially in genetically susceptible patients with subclinical mutations in the *I*_Kr_-encoding *KCHN2*. Few variants in the cardiac Na_V_1.5 Na^+^ channel complex have been associated with diLQTS. We tested whether a novel *SNTA1* (α1-syntrophin) variant (p.E409Q) found in a patient with diLQTS increases late sodium current (*I*_Na-L_), thereby providing a disease mechanism. Electrophysiological studies were performed in HEK293T cells co-expressing human Na_V_1.5/nNOS/PMCA4b with either wild type (WT) or *SNTA1* variants (A390V-previously reported in congenital LQTS; and E409Q); and in adult rat ventricular cardiomyocytes infected with *SNTA1* expressing adenoviruses (WT or one of the two *SNTA1* variants). In HEK293T cells and in cardiomyocytes, there was no significant difference in the peak *I*_Na_ densities among the *SNTA1* WT and variants. However, both variants increased *I*_Na-L_ (% of peak current) in HEK293T cells (0.58±0.10 in WT vs. 0.90±0.11 in A390V, p = 0.048; vs. 0.88±0.07 in E409Q, p = 0.023). In cardiomyocytes, *I*_Na-L_ was significantly increased by E409Q, but not by A390V compared to WT (0.49±0.14 in WT vs.0.94±0.23 in A390V, p = 0.099; vs. 1.12±0.24 in E409Q, p = 0.019). We demonstrated that a novel *SNTA1* variant is likely causative for diLQTS by augmenting *I*_Na-L_. These data suggest that variants within the Na_V_1.5-interacting α1-syntrophin are a potential mechanism for diLQTS, thereby expanding the concept that variants within congenital LQTS loci can cause diLQTS.

## Introduction

Drug-induced long-QT syndrome (diLQTS) is an acquired disorder, most often due to drug block of *I*_Kr_, such as antiarrhythmic agents.[1–3] Recent data suggests that certain individuals have increased susceptibility to diLQTS because of reduced “repolarization reserve” due to subclinical mutations in the two most common congenital long QT syndrome (cLQTS) loci *KCNH2* or *KCNQ1*, both of which encode K^+^ channels.[4, 5] Addition of *I*_Kr_-blocking drugs in these vulnerable populations may underlie increase in action potential (AP) duration through increased late cardiac sodium current (*I*_Na-L_), leading to prolongation of QT interval associated with fatal ventricular tachyarrhythmias.[6] Increased *I*_Na-L_ due to drug-induced decreased phosphoinositide 3-kinase (PI3K) signaling may also contribute to QT prolongation.[7] Variants in *SCN5A*, which encodes the cardiac Na^+^ channel Na_V_1.5 and is the third most common cLQTs locus, however, have been only rarely associated with diLQTS.[8]

α1-syntrophin (SNTA1) encoded by *SNTA1* is a dystrophin-associated protein and a potent regulator of voltage-gated Na^+^ channels. SNTA1 is a component of the Na_V_1.5 channel macromolecular complex through an interaction with the pore-forming α subunit’s C terminus.[9–11] SNTA1 contains 4 protein interacting domains: a postsynaptic density protein-95/disc large/zona occludens-1 (PDZ) domain;[12] two pleckstrin homology (PH1 and PH2) domains;[13] and a syntrophin unique (SU) domain.[14] ^[^^14^^]^The PDZ domain of SNTA1 interacts with a PDZ binding motif comprised of the last 3 amino acids (serine-isoleucine-valine) of the Na_V_1.5 COOH-terminus; and SNTA1 also interacts with the plasma membrane Ca-ATPase (PMCA) 4b, thus forming a complex of all three proteins. This complex can inhibit neuronal nitric oxide synthase (nNOS),[10] reducing NOS-mediated NO production.[15] The physiological and clinical relevance of this interaction is highlighted by the previous identification in a patient with LQTS of a variant (A390V) within the PH2 domain of SNTA1 that led to disrupted binding between SNTA1 and to PMCA4b, thereby relieving inhibition of nNOS and resulting in increased pathogenic *I*_Na-L_ through S-nitrosylation of Na_V_1.5, mediated by local accentuated NO production.[16] The increased *I*_Na-L_ induces cardiac arrhythmias through prolonging AP duration and reducing repolarization reserve.[17, 18]

Previously, no variants in *SNTA1* have been reported in patients with diLQTS. We found a novel *SNTA1* variant (p.E409Q) found in a patient with diLQTS. Thus, we investigated whether this *SNTA1* variant is pathogenic by increasing the *I*_Na-L_. This discovery extends our understanding of how variants in cLQTS loci can increase susceptibility to diLQTS.

## Materials and Methods

### Genetic Analysis

Genetic analysis was obtained as a part of routine care for the patient and was performed in a commercial laboratory after obtaining written informed consent from the family. Consent was also given for further analysis of the biophysical properties of the subsequently identified mutation. Specific written consent is not required by either the Institutional Review Boards or either University of Virginia or Duke University Medical Centers for presentation of a case report.

### Subcloning and adenovirus production

The cDNAs of *SNTA1* (Genebank accession no. NM_003098.2) in the pIRES2EGFP and nNOS (Genebank accession no. NM_052799.1) in pcDNA3.1 were kind gifts from Jonathan C. Makielski (University of Wisconsin, Madison). The PMCA4b (Genebank accession no. AY560895) was subcloned into pcDNA3.1 (Addgene).

The human SNTA1 plasmid vector was mutated by using Quickchange II Site-Directed Mutagenesis (Agilent Technologies, Santa Clara, CA) and the following primers: for A390V-SNTA1, 5´ GTCACCGCAGGAGCTGG**T**TGCCTGGACCCGCCAGC 3´ (forward) and 5´ GCTGGCGGGTCCAGGCA**A**CCAGCTCCTGCGGTGAC 3´ (backward); for E409Q-SNTA1, 5´GCCGCCGAGGGTGTGCAG**C**AGGTGTCTACAGCCTGCAC 3´ (forward) and 5´GTGCAGGCTGTAGACACCT**G**CTGCACACCCTCGGCGGC 3´ (backward). The underlined and bolded nucleotides indicate the variants. These constructs were then subcloned into the pAdRFP adenovirus shuttle vector. Polymerase chain reactions and bacteria transformations were performed according to the manufacturer’s instructions. WT-*SNTA1* and the mutants viruses were generated by using the AdEasy system (Agilent Technologies, Santa Clara, CA).[19] The adenoviral plasmid was packaged in HEK293 cells. The recombinant virus was isolated by multiple freeze/thaw cycles, further amplified and then purified and concentrated using Vivapure AdenoPACK 20 (Sartorius Stedim Biotech, Goettinggen, Germany). The viral titer was determined and used at a multiplicity of infection (MOI) of 50–200. All constructs were confirmed by sequencing.

### HEK293T cell transfection and electrophysiology

HEK293T cells was transfected with tetrodotoxin (TTX)-sensitive Na_V_1.5, nNOS, PMCA4b and the pIRES2EGFP plasmids vector expressing either human WT or *SNTA1* mutants (A390V and E409Q) at a ratio of 4:4:4:1 at a confluency of 60% using Lipofectamine 2000 (Life Technologies). The cells were incubated at 37°C for 2 to 3 days before use. Transfected cells were identified by green fluorescent protein (GFP).

Na^+^ currents were recorded using the whole-cell voltage-clamp technique at room temperature (20–22°C) 48–72 hours after transfection, as previously described.[20] The bath solution containing (in mM, 300 mosm): NaCl 120, TEA-Cl 20, KCl 5.4, CaCl2 1.8, MgCl2 1, HEPES 10, D(+)-glucose 10, pH 7.4 adjusted with NaOH. The pipette solution containing (in mM, 290–295 mosm): CsCl 50, CsF 30, L-aspartic acid 50, EGTA 5, HEPES 10, NaCl 10, pH 7.3 adjusted with CsOH. Osmolarity was adjusted with sucrose for all solutions. Electrode resistance ranged from 2 to 4 MΩ. Standard step-pulse voltages were generated with Axopatch 200B amplifier using pClamp 9.0 software (Axon Instruments). Currents were filtered at 5 kHz and digitalized using an analog-to-digital interface (Digidata 1322A, Axon Instruments). To measure current amplitude data and voltage-dependence of steady-state activation, currents were elicited by a 50 ms pulse from a holding potential of -120 mV to test potentials between -100 mV and +60 mV in 5 mV increments. Current density (pA/pF) was calculated by normalization to cell capacitance. Conductance (G) was calculated by dividing the peak current for each voltage step by the driving force (*V*_m_-*V*_r_), then normalizing to the peak conductance (*G*_max_). The data were fitted with the Boltzmann function of the form *G*/*G*_max_ = 1/{1+exp[(*V*_1/2_-*V*_m_)/*k*]} in which *V*_1/2_ is the voltage at which half of Na_V_1.5 channels are activated, *k* is the slope factor, and *V*_m_ is the membrane potential. Standard two-pulse protocols were used to generate the steady-state inactivation curves: from the holding potential -120 mV, cells were stepped to 500-ms preconditioning potentials varying between -140 mV and -20 mV (prepulse), followed by a 20 ms test pulse to -40 mV. Currents (*I*) were normalized to *I*_max_ and fit to a Boltzmann function of the form *I*/*I*_max_ = 1/{1+exp[(*V*_m_-*V*_1/2_)/*k*]} in which *V*_1/2_ is the voltage at which half of Na_V_1.5 channels is inactivated, *k* is the slope factor, and Vm is the membrane potential. Recovery from inactivation was analyzed by fitting data with the two exponential function: *I(t)*/*I*_max_ = A_f_×[1–exp(–*t*/τ_f_)]+As× [1–exp(–*t*/τ_s_)], where values for A and τ refer to amplitudes and time constants, respectively. *I*_Na-L_ was determined with 200-ms depolarization from -120 m V to -10 m V as the average between 145–150 ms after the initiation of the depolarization and reported as a percentage of peak current following digital subtraction of currents recorded in the presence and absence of 1 μmol/L TTX (AbcamBiochemicals) as previously described.[21] Curve fitting and data analysis were performed using Clampfit 10.5 software (Axon Instruments) and Origin 9.1 (Originlab Corporation).

### Isolation, culture, and adenoviral infection of adult rat ventricular myocytes

Animals were handled according to National Institutes of Health’s *Guideline for the Care and Use of Laboratory Animals*. The study was approved by Duke University Animal Care and Welfare Committee. Cardiomyocytes were isolated from 6- to 8-week-old Sprague-Dawley rats (Charles River Laboratories, Wilmington, MA) and cultured as described previously.[22, 23] The origin of Sprague-Dawley rats was to SASCO from ARS/Sprague Dawley in 1979/to Charles River in 1996. Rats were housed in cages in a sterilized room in which temperature and humidity ranges are controlled appropriate for the rats (20–26°C, 30–70% humidity; 10–15 fresh-air changes per hour provided by ventilation). A time-controlled lighting system was used to ensure a regular diurnal cycle, and timer performance is checked periodically to ensure proper cycling. Animals were fed palatable, non-contaminated, and nutritionally adequate food daily based on comprehensive treatments of the nutrient requirements of laboratory animals prepared by the National Research Council Committee on Animal Nutrition. The rats had access to potable, uncontaminated drinking water. Animals were anesthetized with tribromoethanol (250 mg/kg, intraperitoneal injection) and anti-coagulated with heparin following Duke University Animal Care & Use Program guidelines for systemic anesthetics in rats. We confirmed that the rats were completely anesthetized prior to removing their hearts. Hearts were removed and the aorta was cannulated to retrogradely perfuse the heart using a Langendorff apparatus (Radnoti Glass Technology, Inc) for about 10 minutes. The heart were first perfused with basal solution containing (in mM, from Sigma unless otherwise specified): NaCl 112, KCl 5.4, NaH_2_PO4∙H_2_O 1.7, NaHCO_3_ 4.2, MgCl∙6H_2_O 1.63, HEPES 20.04, D(+)-glucose 5.4, taurine 10, L-carnitine 2, creatinine 2.3, glucose 5.4, taurine 10, L-carnitine 2, creatine 2.3, 2,3-butanedione monoxime (BDM) 10. After five minutes, the solution was switched to basal solution plus 150 u/ml Collagenase Type II (Worthington) and the heart was perfused until it was soft and boggy. The heart was then taken down from the Langendorff. Both ventricles were minced into small pieces, and then triturated in enzyme solution until all cell clumps were broken. The solution was filtered through sterile 190 μm nylon mesh and centrifuged at 300 rpm for 2 minutes. The cells were resuspended in perfusion solution with bovine serum albumin (BSA) at 5 mg/ml to quench the enzyme. Calcium tolerance was performed by gradually adding CaCl_2_ to a final concentration of 1 mM. For culture, cells were plated on laminin coated coverslips in plating medium of Minimal Essential Medium (MEM) with Earle’s Salts and L-glutamine, 10 mM BDM, 5% fetal bovine serum (Life Technologies) and 1% penicillin/streptomycin. After cells had adhered to the plates, the cells were washed once. Virus was resuspended in culture medium and the plating medium changed to culture medium into which the proper adenovirus had been added. Culture medium contained MEM with Earle’s Salts and L-glutamine, bovine serum albumin 0.1 mg/ml, BDM 10 mM, 1X insulin-selenium- transferrin supplement (Gibco), creatine 5 mM, taurine 5 mM, L-carnitine 2 mM, and blebbistatin 25 μM (Toronto Research Chemicals). All solutions were oxygenated in 95% O_2_/5% CO_2_ for at least 30 minutes. Cells were checked for RFP fluorescence 36–48 hours post infection. Rod-shaped, striated cells were analyzed for electrophysiology

### Cardiomyocyte electrophysiology

Na^+^ currents were recorded by using the whole-cell voltage clamp technique in cardiomyocytes, as described previously.[22, 23] Voltage-clamp experiments were performed at room temperature (22–24°C), 36–48 hours after infection of adult cardiomyocytes with adenovirus. Bath (Tyrode) solution contained (in mM): NaCl 140, KCl 5.4, CaCl_2_ 1, MgCl_2_∙6H_2_O 1, HEPES 5, glucose 10, pH 7.2 adjusted with NaOH. Once the cell was ruptured, solution was quickly changed to recording solution containing (in mM): NaCl 20, HEPES 20, CsCl 55, CaCl_2_ 1, MgCl_2_ 1, CsOH 10, 4-aminopyridine 2, D(+)-glucose 10, CdCl_2_ 0.5, TEA-Cl 50, pH 7.35 adjusted with HCl. Internal solution contained (in mM): NaCl 5, CsF 120, HEPES 5, EGTA 10, GTP-Na sulfate 0.5, TEA-Cl 20, pH 7.35 adjusted with CsOH. Osmolarity was adjusted to ~300 mOsm with sucrose for all solutions. Recordings were filtered at 5 kHz and digitally sampled at 20 kHz. The pulse protocol cycle time was 3 seconds to ensure full Na^+^ channel recovery. Current amplitude data for each cell were normalized to its cell capacitance (current density, pA/pF). To determine the voltage-dependence of steady-state activation, currents were elicited by a 50 ms pulse from a holding potential of -120 mV to test potentials between -100 mV and +60 mV in 5 mV increments. The sodium conductance (G) was calculated by dividing the peak current for each voltage step by the driving force (*V*_m_-*V*_r_) then normalized to the peak conductance (*G*_max_). Data were fitted with the Boltzmann relationship, *G*/*G*_max_ = 1/{1+exp[(*V*_1/2_-*V*_m_)/*k*]} in which *V*_1/2_ is the voltage at which half of Na_V_1.5 channels is activated, *k* is the slope factor and *V*_m_ is the membrane potential. Standard two-pulse protocols were used to generate the steady-state inactivation curves: from the holding potential -120 mV, cells were stepped to 500-ms preconditioning potentials varying between -140 mV and -20 mV (prepulse), followed by a 20 ms test pulse to -40 mV. Currents (*I*) were normalized to Imax and fit to a Boltzmann function of the form *I*/*I*_max_ = 1/{1+exp[(*V*_m_-*V*_1/2_)/*k*]} in which *V*_1/2_ is the voltage at which half of Na_V_1.5 channels is inactivated, *k* is the slope factor and *V*_m_ is the membrane potential. Recovery from inactivation was analyzed by fitting data with the two exponential function: *I(t)*/*I*_max_ = A_f_×[1–exp(–*t*/τ_f_)]+As× [1–exp(–*t*/τ_s_)], where values for A and τ refer to amplitudes and time constants, respectively. *I*_Na-L_ was determined with 200-ms depolarization from -120 m V to -40 m V as the average between 190–200 ms after the initiation of the depolarization and reported as a percentage of peak current following digital subtraction of currents recorded in the presence and absence of 30 μmol/L TTX (Abcam Biochemicals) as previously described. The specific groups were blinded to an investigator when the measurements. These data were measured Curve fitting and data analysis were performed using Clampfit 10.5 software (Axon Instruments) and Origin 9.1 (Originlab Corporation).

### Statistical analysis

Results are expressed as mean ± standard error of mean (SEM). Statistical significance of differences between the groups was assessed using Student’s *t*-test. For experiments with deviations from normality, the nonparametric Mann-Whitney *U* test was used for comparison. Values of p≤0.05 were considered statistically significant. Statistical analyses were carried out using SPSS Statistics software version 17.0 (IBM, Armonk, NY).

## Results

### Case report

**Fig 1:**
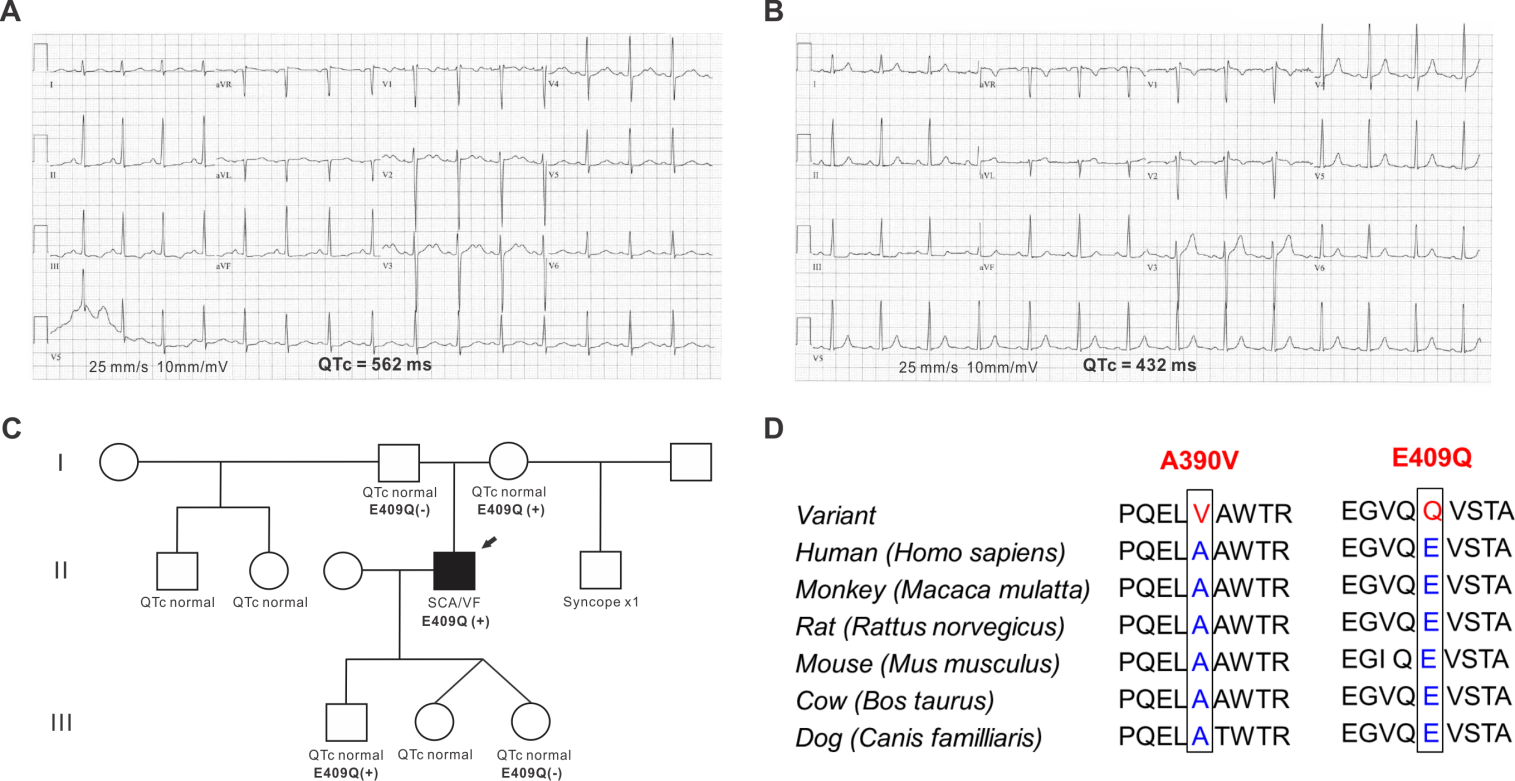
A drug-induced long-QT syndrome patient with the p.E409Q variant in SNTA1. (A) A 12-lead resting electrocardiogram (ECG) from the proband during the post-arrest period showed markedly prolonged QT intervals. The QTc was 562 ms, and the notched T wave was noted. (B) The QTc interval was normalized and the T wave notch disappeared 2 months after aborted sudden cardiac death. (C) Pedigree of the family. An arrow is the proband with p.E409 variant. (D) Sequence conservation across species for A390V and E409Q versus normal in SNTA1. SCA, sudden cardiac arrest; and VF, ventricular fibrillation.

A 36 year old previously healthy man with no family history of sudden cardiac death or LQTS suffered a witnessed cardiac arrest while exercising. At the time of his arrest his medications included amitriptyline, pseudoephedrine, and famotidine. His initial rhythm was ventricular fibrillation from which he was successfully resuscitated with an external countershock. Coronary angiography showed no evidence of coronary artery disease. Cardiac MRI showed an EF of 65% with no evidence of right ventricular dysplasia. ECGs in the post-arrest period were notable for prolonged QT intervals (repeated QTc measurements > 480 ms; the longest was 597 ms) (Fig 1A). ECGs obtained in the month after the arrest and in all follow up visits showed normal QT intervals (all QTc measurements <440 ms) after discontinuation of amitriptyline, pseudoephedrine, and famotidine (Fig 1B).

The patient was prescribed β-blocker therapy and underwent implantable cardioverter-defibrillator (ICD) implantation. He received one ICD shock for polymorphic ventricular tachycardia after re-initiation of vigorous exercise while he was only intermittently compliant with β-blocker therapy.

### Genetic testing and sequence homologies for the *SNTA1* variants

Genetic testing with a 30-gene arrhythmia panel revealed a novel heterozygous missense variant (c.1225 G>C; p.Glu409Gln, p.E409Q) in *SNTA1*. The pedigree is shown in Fig 1C. This variant is within a residue highly conserved across species (Fig 1D), and is near to the originally reported A390V *SNTA1* variant associated with LQTS,[16] which we used as a positive control. The p.E409Q *SNTA1* variant is not present in normal population databases including the Exome Aggregation Consortium (ExAC),[24] the NHLBI ESP Exome Variant Server (EVS),[25] and the 1000 Genomes Project.[26] Multiple in-silico analyses predicted the SNTA1 variant, p.E409Q, to be pathogenic: PolyPhen-2 (prediction = probably damaging, score = 1.000),[27] MutationTaster2 (prediction = disease-causing, probability value = 0.999),[28] and SIFT (prediction = damaging, score = 0).[29]

### Biophysical properties of Na_V_1.5 Co-expressed with PMCA4b, nNOS, and SNTA1

**Fig 2:**
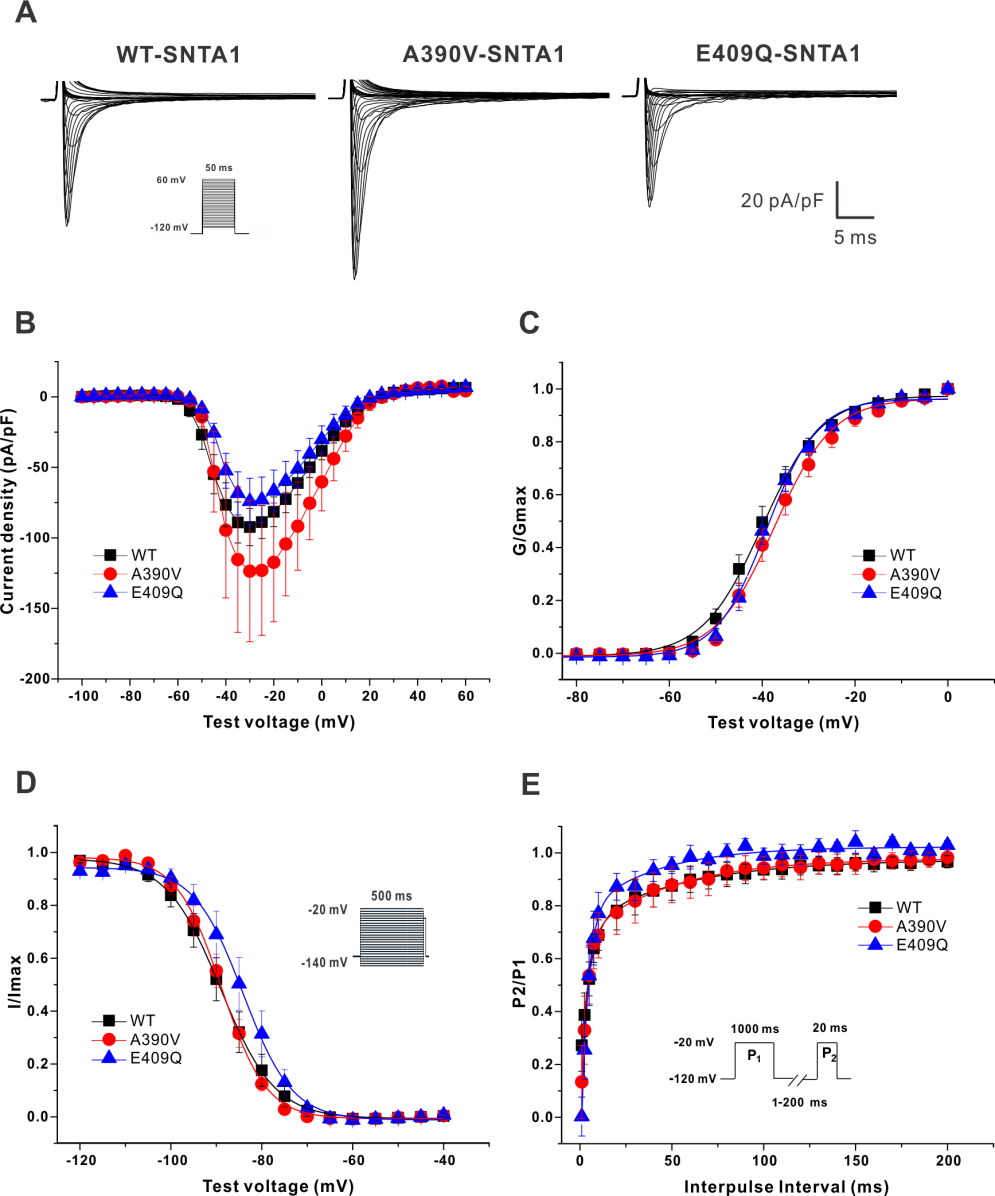
Electrophysiological data of Na_V_1.5 in HEK293T cells coexpressing PMCA4b, nNOS, and either WT or SNTA1 mutants. (A) Representative traces of inward Na^+^ current for the 3 groups tested. (B) I-V curve. (C) Activation (G/Gmax). (D) Inactivation (I/Imax). (E) Recovery (P2/P1).

We recorded voltage-gated Na^+^ currents in HEK293T cells, in which we transiently co-expressed components of the Na^+^ channel macromolecular complex necessary for regulation by *SNTA1*. Specifically, we expressed human Na_V_1.5 (with a C373Y mutation rendering the channel sensitive to TTX), nNOS, and PMCA4b with WT SNTA1 or either of the two SNTA1 mutants. Previous studies have shown that the TTX-sensitive mutation does not affect any permeation properties.[30] Table 1 shows the summary data for the 3 groups. Representative traces of whole-cell currents (Fig 2A) and I-V curves (Fig 2B) show that neither mutant affected peak *I*_Na_ current density nor the kinetics of activation compared to WT-SNTA1 (Fig 2C). The *k* of inactivation was significantly reduced only in E409Q-SNTA1 (p<0.001). There was no significant difference in rate of fast recovery from inactivation using a two-pulse protocol among WT and the mutants, but the rate of slow recovery was significantly prolonged in A390V-SNTA1 compared to WT-SNTA1 (p = 0.035) (Fig 2D). Focusing only on the mutants, there was no significant difference in all parameters of activation, inactivation and recovery (Table 1).

**Table 1:** Summary of electrophysiological data in the HEK293T cells.

	WT-SNTA1	A390V-SNTA1	E409Q-SNTA1
Peak *I*_Na_ at -30mV, pA/pF	-92.3±13.6 (17)	-123.6±53.0 (9)	-74.1±17.3 (10)
Activation			
*V*_1/2_, mV	-40.9±1.5 (17)	-37.2±2.1 (9)	-39.8±1.4 (10)
*k*, pA/mV	4.3±0.2 (17)	5.0±0.7 (9)	4.1±0.3 (10)
Inactivation			
*V*_1/2_, mV	-88.8±2.1 (12)	-88.7±1.4 (8)	-84.2±2.3 (9)
*k*, pA/mV	4.1±0.2 (12)	3.6±0.4 (8)	3.4±0.1* (9)
Recovery (P2/P1)			
tau fast recovery, ms	5.32±2.53 (7)	2.45±0.56 (6)	3.47±0.75 (7)
tau slow recovery, ms	18.05±4.47 (7)	36.18±5.79* (6)	22.95±5.43 (7)
*I*_Na-L,_ %	0.581±0.097 (8)	0.899±0.110* (8)	0.883±0.065* (9)

The number of cells analyzed for each parameter is in parentheses; and *I*_Na-L,_, late Na current.

* P-value < 0.05 versus WT-SNTA1.

### *SNTA1* variants increase late *I*_Na_ in HEK293T cells

**Fig 3:**
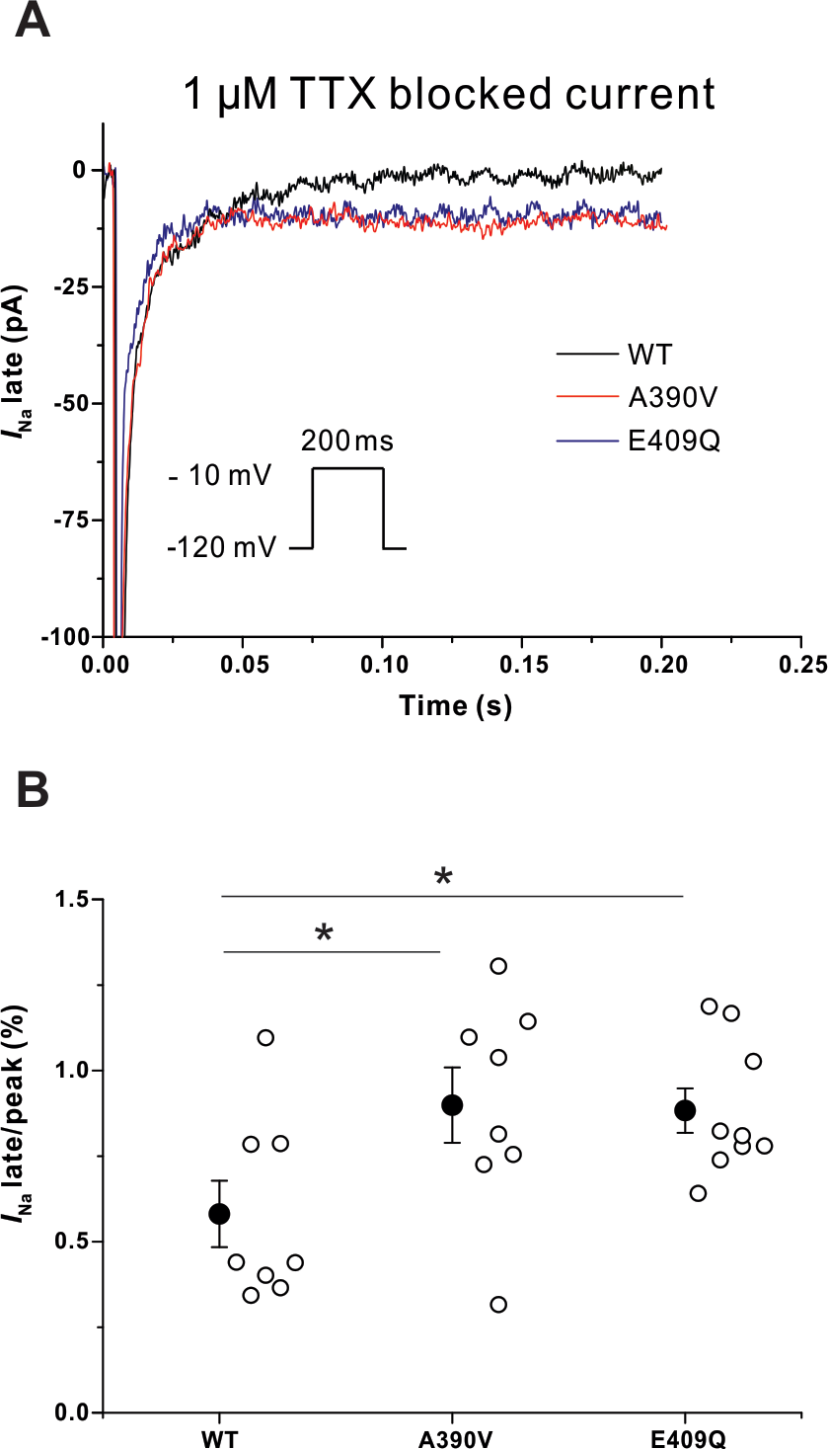
Late Na^+^ current in HEK293T cells. (A) Representative late Na^+^ currents with WT and the SNTA1 variants (Fig 3A). (B) Both A390V-SNTA1 and E409Q-SNTA1 significantly increased *I*_Na-L_ in HEK293T cells compared to that of WT-SNTA1. * P<0.05 versus WT-SNTA1. *I*_Na_ indicates sodium current; TTX, tetrodotoxin. Mean and standard error of mean (SEM) are shown in the graph.

The *I*_Na-L_ was measured using a long depolarization pulse (200 ms at -10 mV from a holding potential of -120 mV) and background was subtracted by administration of TTX (1 μM). Representative traces are shown in Fig 3A and the data are summarized in Table 1. *I*_Na-L_ (% of peak current) was significantly increased with both mutants compared to WT-SNTA1 (0.58±0.10 in WT vs. 0.90±0.11 in A390V-SNTA1, p = 0.048; vs. 0.88±0.07 in E409Q-SNTA1, p = 0.023) (Fig 3B). There was no significant difference in *I*_Na-L_ between A390V-SNTA1 and E409Q-SNTA1 (p = 0.903).

### Biophysical properties of sodium currents in adult rat cardiomyocytes

**Fig 4:**
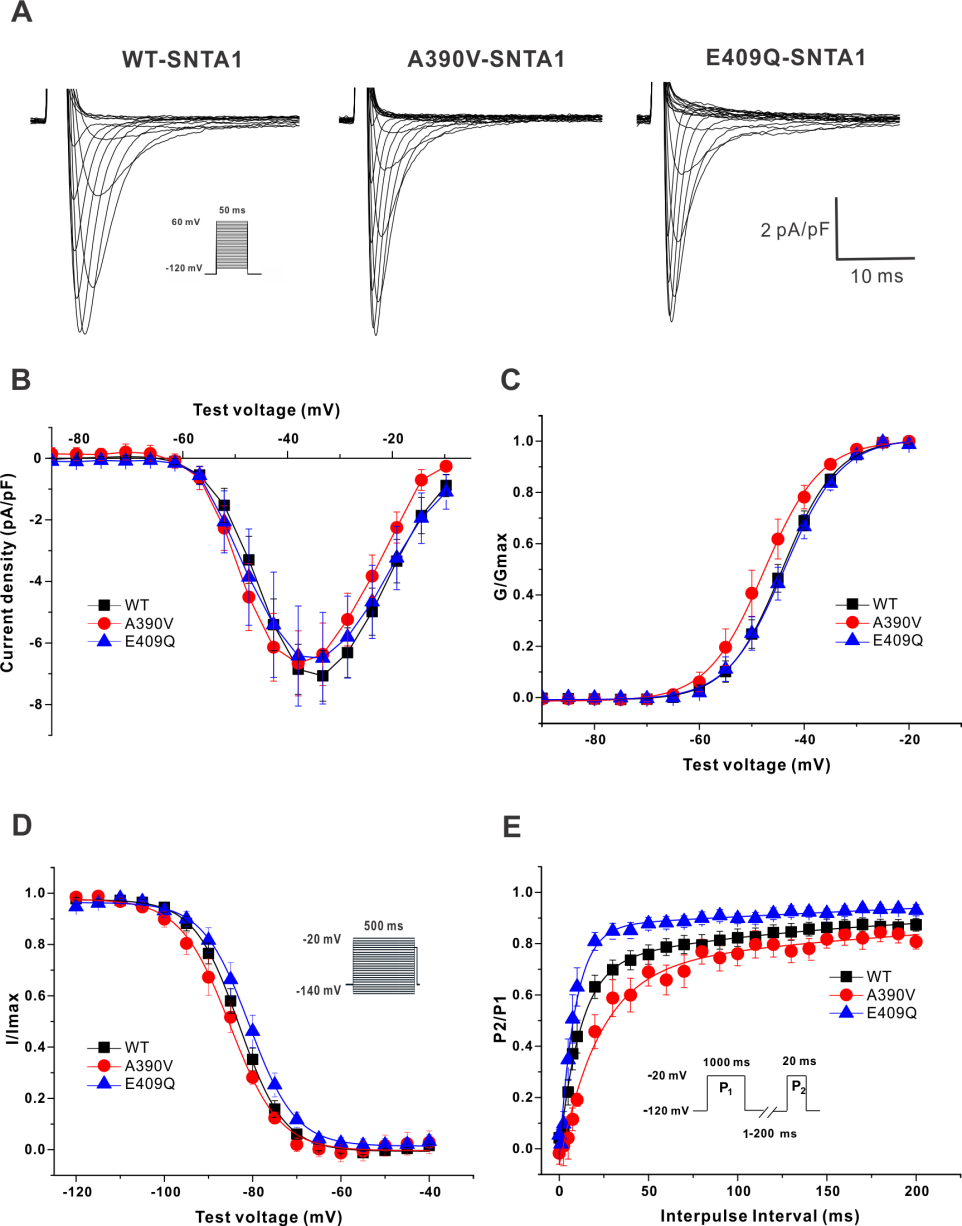
Electrophysiological data of Na+ current in adult rat cardiomyocytes which were infected with either WT or SNTA1 mutants. (A) Representative traces of inward Na^+^ current for the 3 groups tested. (B) I-V curve. (C) Activation (G/Gmax). (D) Inactivation (I/Imax). (E) Recovery (P2/P1).

To confirm these results, we also recorded voltage-gated Na^+^ currents in cultured adult rat cardiomyocytes 36–48 h after they were infected with WT or either of the two SNTA1 variants. Because the patient is heterozygous for the *SNTA1* variant, we expressed the WT or the variants without knocking down the endogenous *Snta1* in the rat cardiomyocytes, thus more accurately recapitulating the patient’s condition in which the WT and variant were both present. Table 2 shows the summary data for the 3 groups. Representative traces of whole-cell currents were shown in Fig 4A. I-V curves (Fig 4B) show that neither mutant affected peak *I*_Na_ current density (WT-SNTA1 vs. A390V-SNTA1, p = 0.895; vs. E409Q-SNTA1, p = 0.929). In addition, there was no significant difference in the current voltages between the variants (A390V-SNTA1 vs. E409Q-SNTA1, p = 0.994). The kinetics of activation and steady-state inactivation of WT and mutants were shown in Fig 4C and 4D. There was no significant difference in kinetics of activation and steady-state inactivation between WT and either of the SNTA1 variants. Compared to WT-SNTA1, the rates of recovery from inactivation appeared to be slightly delayed in E409Q-SNTA, but there was no statistical significance (Fig 4E).

**Table 2:** Summary of electrophysiological data in the adult rat cardiomyocytes.

	SNTA1-WT	SNTA1-A390V	SNTA1-E409Q
Peak *I*_Na_ at -40mV, pA/pF	-6.8±0.8 (13)	-6.6±1.1 (7)	-6.6±1.6 (14)
Activation			
*V*_1/2_, mV	-44.5±1.2 (13)	-47.9±1.8 (7)	-44.1±1.4 (14)
*k*, pA/mV	4.4±0.1 (13)	4.3±0.2 (7)	4.0±0.2 (14)
Inactivation			
*V*_1/2_, mV	-83.1±1.1 (12)	-83.6±0.6 (7)	-81.3±1.5 (10)
*k*, pA/mV	4.8±0.2 (12)	4.9±0.3 (7)	4.7±0.2 (10)
Recovery (P2/P1)			
tau, ms	10.9±1.6 (11)	25.5±8.4 (5)	7.1±1.3 (8)
tau fast recovery, ms	8.8±1.7 (11)	23.3±8.6 (5)	6.8±1.4 (8)
tau slow recovery, ms	119.7±20.6 (11)	97.2±42.7 (5)	89.3±31.6 (8)
*I*_Na-L,_ %	0.49±0.14 (8)	0.94±0.23 (5)	1.12±0.24* (6)

The total number of rats used in the study was 11. The experiments were performed on cells derived from multiple independent isolations. Some cells were recorded from a single rat isolation. The number of cells analyzed for each parameter is in parentheses; and *I*_Na-L,_, late Na current.

* P-value < 0.05 versus SNTA1-WT.

### *SNTA1* variants increase *I*_Na-L_ in adult rat cardiomyocytes

**Fig 5:**
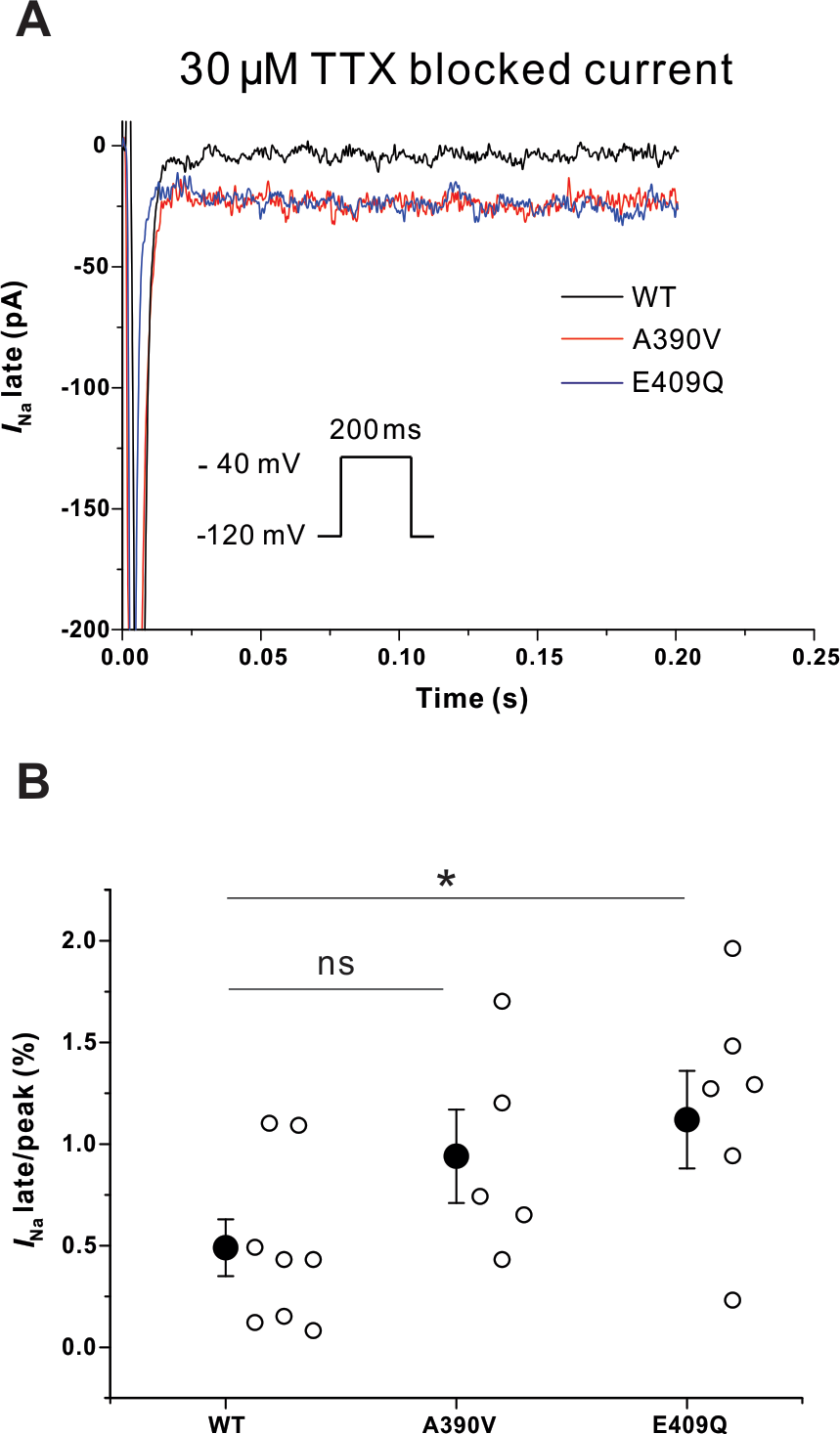
Late Na^+^ current in adult rat cardiomyocyte infected with adenoviruses expressing either the WT or one of the two *SNTA1* mutants. (A) Representative late Na^+^ currents in with WT and the *SNTA1* variants (Fig 5A). (B) E409Q-SNTA1 significantly increased *I*_Na-L_ compared to that of WT-SNTA1. * P<0.05 versus WT-SNTA1. *I*_Na_ indicates sodium current; TTX, tetrodotoxin. Mean and standard error of mean (SEM) are shown in the graph.

The *I*_Na-L_ was measured using a long depolarization pulse (200 ms at -40 mV from a holding potential of -120 mV) and background was subtracted after TTX (30 μM) was applied to the bath. Representative traces are shown in Fig 5A and the data are summarized in Table 2. Compared to WT-STNA1, *I*_Na-L_ (% of peak current) was significantly increased with E409Q mutant, but not the A390V mutant (0.49±0.14 in WT-SNTA1 vs. 0.94±0.23 in A390V-SNTA1, p = 0.099; vs. 1.12±0.24 in E409Q-SNTA1, p = 0.019) (Fig 5B). There was no significant difference in *I*_Na-L_ between A390V-SNTA1 and E409Q-SNTA1 (p = 0.903).

## Discussion

### Principal findings

This is the first report to our knowledge of a *SNTA1* variant in a patient with diLQTS and a first demonstration that SNTA1 affects *I*_Na-L_ in adult rat cardiomyocytes. We provide functional data demonstrating a gain-of-function for a novel *SNTA1* variant, p.E409Q, leads to diLQTS by augmenting *I*_Na-L_. Overall, the effects of the p.E409Q variant on Na^+^ currents are generally similar to the effects of the p.A390V variant that was the original LQT mutation described in *SNTA1*.^16^

### Syntrophin mutation and late sodium currents

**Fig 6:**
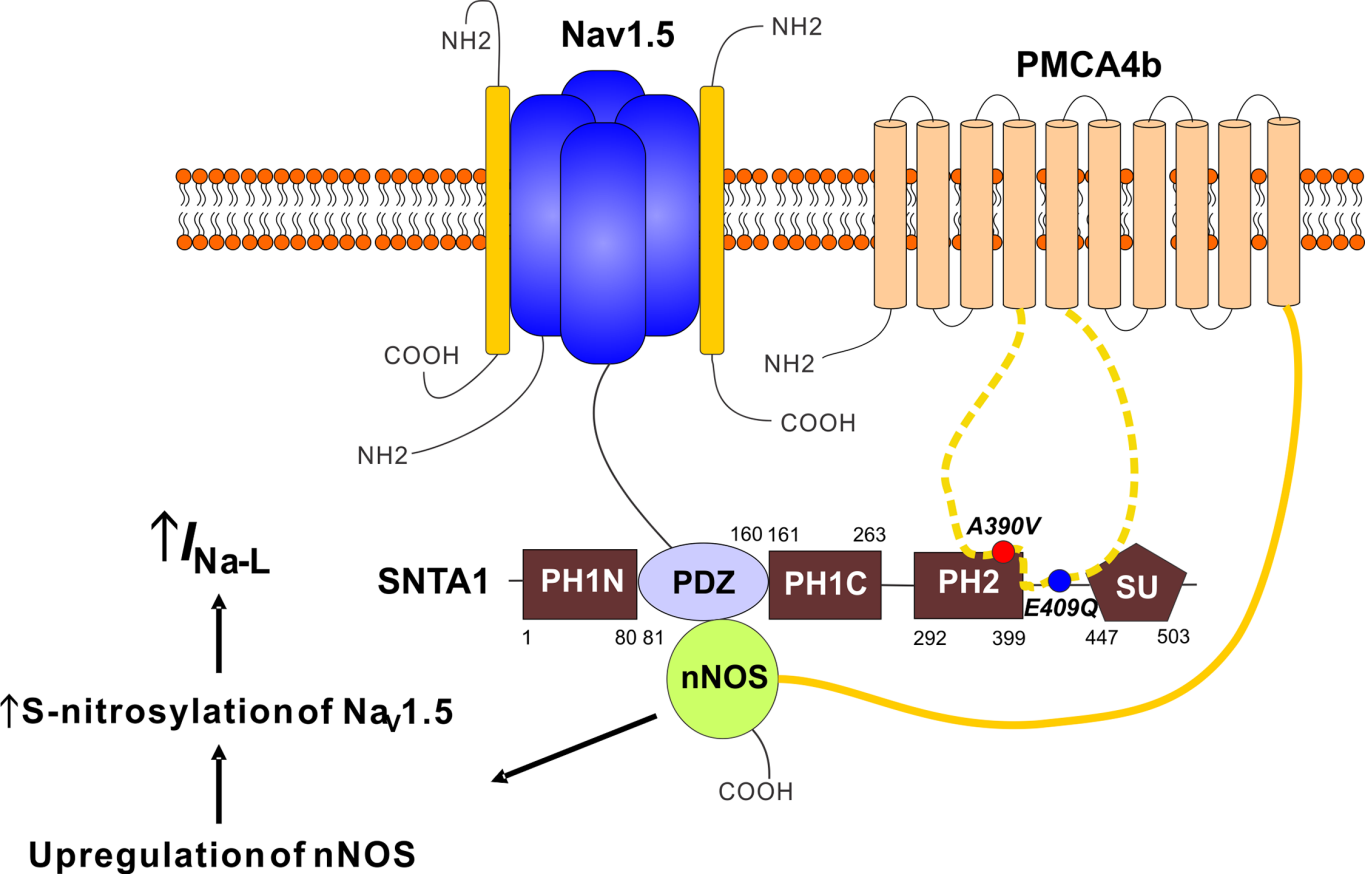
Syntrophin mutation and late sodium current. A schematic diagram shows variants in SNTA1 lead to disrupted binding to PMCA4b and released inhibition of nNOS, resulting in increased *I*_Na-L_ through S-nitrosylation of Na_V_1.5.

SNTA1 is now a well-established a Na_V_1.5 channel interacting protein (NaChIP) in complex with nNOS and PMCA4b.[31] Mutations lead to gain-of-function modulations of Na_V_1.5 (increased *I*_Na-L_) and cLQTS. Ueda et al. reported a missense mutation (p.A390V) within the PH2 domain of SNTA1 disrupted its binding with PMCA4b, thereby disinhibiting nNOS, which caused S-nitrosylation of Na_V_1.5 and a resultant increase in *I*_Na-L_.[16] The novel variant tested here, p.E409Q lies outside of the PH2 domain. Thus, the data showing that p.E409Q affects Na_V_1.5 currents similarly to p.A390V in both cardiomyocytes, and in a heterologous system in which all the key components of the macromolecular complex are present, suggest that E409Q affects PMCA4b interaction similarly to A390V. We conclude, therefore, that the binding site for PMCA4b must extend further towards the C-terminus on PMCA4b than the PH2 domain or that the E409Q variant affects the PH2 binding domain allosterically. A schematic depicting this proposed interaction and the consequent mechanism for increased *I*_Na-L_ is shown in Fig 6.

Recent cohort-based studies in patients with cLQTS reported that increased *I*_Na-L_ due to mutations in *SNTA1* is a pathogenic mechanism for cLQTS and a subset of channelopathic sudden infant death syndrome (SIDS),[32, 33] and the mutations also represented gain-of-function of *I*_Na_. Our data also showed that two S*NTA1* variants increase *I*_Na-L_ without causing a significant difference in peak *I*_Na_ compared to wild type SNTA1.

The absence of an effect on peak current (recorded in the absence of TTX) is an important result from our studies that helps add confidence to our measurements of *I*_Na-L_, which is a generally challenging analysis given its relatively small amplitude. Because peak current was unchanged, it is unlikely that observed increase in the small amplitude *I*_Na-L_ (for which we used TTX to effectively isolate *I*_Na-L_ from other currents) simply reflects a change in the peak current. Rather, it likely reflects a true change in inactivation properties of the channel as influenced by the channel’s macromolecular complex. Two additional factors add to our confidence in accurately measuring this small amplitude *I*_Na-L_. First, we performed the experiments in both HEK cells and myocytes, and obtained consistent results. Second, the analysis of the *I*_Na-L_ amplitude was performed while blinded to the *SNTA1* genotype, thus avoiding any unintentional bias.

While generally consistent with previous studies of *SNTA1* variants, our results are slightly different in a subset of electrophysiologic parameters. In HEK293T cells, the E409Q mutant showed a reduced *k* of inactivation whereas previous studies demonstrated a reduced *V*_1/2_ but not *k* of inactivation. In addition, we tested the electrophysiologic properties of SNTA1 mutants in adult rat cardiomyocytes using adenoviral expression. In our data, the recovery from inactivation appeared to be different in comparing HEK293T cells and cardiomyocytes. The reasons for this are not known, but it could be that there are additional regulatory components in the native myocytes that are regulating the interaction of SNTA1 and SCN5A. Alternatively, the stoichiometry of the transfected/infected components may be different between the two systems. Nevertheless, these data, querying the roles of wild type and mutant SNTA1 proteins in their native environment and in an adult cardiomyocyte add new information beyond the previous studies.

### *SNTA1*: A novel susceptibility gene for diLQTS

The voltage gated cardiac sodium channel is known to be responsible to a subgroups of LQTS (LQT3).[34] Other subgroups of LQTS (LQT9, LQT10 and LQT12) also affect the β subunit of Na_V_1.5 or the NaChIPs, such as caveolin-3 and SNTA1, leading to increased *I*_Na-L_.[16, 35, 36] Our patient was diagnosed with diLQTS, an acquired or iatrogenic disease, which has been described most commonly as caused by repolarization abnormalities due to block of potassium channels.[3] The notched T wave shown in Fig 1A is consistent with the pattern observed after *I*_Kr_ block.[37] *I*_Kr_ (hERG channel) blocking drugs, prokinetics or antiarrhythmic drugs, decrease a patient’s “repolarization reserve” and then can prolong the QT interval.[4] The patient studied here, was exposed to three drugs (the tricyclic antidepressant amitriptyline; pseudoephedrine; and the H_2_-receptor antagonist famotidine) before his episode of aborted sudden death, two of which (amitriptyline and pseudoephedrine) are known to cause QT interval prolongation. Thus, we conclude that this episode of diLQTS resulted from an underlying genetic susceptibility due to a mutation in a cLQTS locus (*SNTA1*) exacerbated by a reduction in the repolarization reserve caused by amitriptyline and pseudoephedrine. Consistent with our hypothesis, Itoh et al. reported that diLQTS had a similar positive mutation rate compared to cLQTS and QT prolongation by *I*_Kr_-blocking agents was excessive in dLQTS subjects with the underlying genetic background.[38] They concluded that certain individuals may have increased susceptibility to diLQTS because of reduced “repolarization reserve” due to subclinical mutations in the cLQTS loci *KCNH2* or *KCNQ1*, both of which encode K^+^ channels. Our results expand this concept to include susceptibility due to a variant in a *SNTA1*. In previous reports regarding pathologic *SNTA1* variants, the affected patients presented with a cLQTS phenotype in contrast to the index patient here, who had an aborted sudden death event in the setting of medications and who showed normal QT intervals on ECGs after discontinuation of the drugs. In addition, the proband’s family members carrying the same variants also showed normal QTc interval (Fig 1C) and no evidence of symptoms. A reasonable explanation for these observations is that these individuals might harbor genetic modifier protecting from the cLQTS, but in the absence of whole exome sequencing we cannot be certain.

The connection between a reduced repolarization reserve via K^+^ channel mutations and susceptibility due to a mutation in the Na_V_1.5 macromolecular complex has been suggested by several recent reports. Wu et al. demonstrated that endogenous late *I*_Na_ contributed to the reverse rate dependence of *I*_Kr_ inhibitor-induced increases in action potential duration and beat-to-beat variability of repolarization, which are proarrhythmic.[6] Recently, Yang et al. showed some *I*_Kr_ blockers with torsades liability, such as dofetilide, increase *I*_Na-L_ through inhibition of phosphoinosotide 3-kinase (PI3K) pathway.[5] Exposure to dofetilide generated arrhythmogenic afterdepolarizations and ≥ 15-fold increases in *I*_Na-L_, and a downstream effector for the PI3K pathway inhibited these effects.^5^ Many anti-cancer drugs that target the PI3K signaling have been developed, and inhibition of the PI3K pathway has been reported as the cause of a diLQTS in which alterations in several ion currents contribute to arrhythmogenic drug activity.[39] Lin and Cohen et al demonstrated cardiac myocytes of mice with diabetes exhibited an increase in action potential duration (APD) by altering *I*_Na-L_, which was reversed by expression of constitutively active PI3K.[40] While the specific drugs used by the proband in this study have not been shown to affect PI3K, neither were drugs such as dofetilide until these recent studies. Thus, an alternative mechanism that could explain this proband’s arrhythmia is through inhibition of the PI3K pathway or that the *SNTA1* variant renders the channel complex more susceptible to changes in PI3K metabolites.

Nevertheless, those studies firmly identify the Na_V_1.5 cardiac sodium channel as an important mediator of diLQTS and fit with prior studies, such as Makita et al., which observed that subclinical mutations (L1825P) in the LQTS-related gene *SCN5A* might predispose certain individuals to diLQTS when treated with the prokinetic drug cisapride, a K^+^ channel blocking agent.[41] S1103Y, a common *SCN5A* variant, has been associated with a predisposition to abnormal cardiac repolarization and acquired arrhythmia when cardiac potassium channel blocking medications, such as amiodarone, were administered.[8] [42] In this context, our data are consistent with those reports and extend the model beyond Na_V_1.5 mutations to NaChIPs.

Beyond diLQTS and cLQTS, *I*_Na-L_ in cardiomyocytes can be increased by acquired conditions such as heart failure.[42] Whether variants in *SNTA1* increase the risk of arrhythmias in heart failure patients has not been tested, but would fit with a previous study showing that the S1103Y polymorphism in *SCN5A* confers an increased risk of arrhythmogenesis in patients with heart failure.[43] In summary, our study demonstrated that a novel *SNTA1* variant, p.E409Q, increased the *I*_Na-L_ and is a potential mechanism for acquired lethal ventricular arrhythmias.

### Clinical implications

This study may provide additional motivation not only for genetic screening in patients experiencing diLQTS, but it suggests that a broad panel of cLQTS loci should be tested—not only *KCNQ1* or *KCHN2*. Identification of variants in cLQTS loci in a patient suffering from diLQTS may be motivation for cascade screening and consequent advice to affected family members to avoid known QT prolonging drugs. Further, for those patients suffering diLQTS in the setting of *SNTA1* or variants in other NaChIPs, treatment with agents targeting *I*_Na-L_ such as ranolazine may be a reasonable strategy,[44] particularly in cases when concomitant treatment with a QT prolonging agent is deemed necessary.

## Conclusions

In conclusion, we demonstrated a novel *SNTA1* variant, E409Q-SNTA1, leads to diLQTS by augmenting *I*_Na-L_. These data suggest the variant of the Na_V_1.5-interacting α1-syntrophin is a potential mechanism for the genetic susceptibility in patients with diLQTS, thereby expanding, beyond K^+^ channel loci, the concept that variants within cLQTS can cause diLQTS.
